# Dynamic Changes in Progesterone Concentration in Cows’ Milk Determined by the At-Line Milk Analysis System Herd Navigator^TM^

**DOI:** 10.3390/s20185020

**Published:** 2020-09-04

**Authors:** Ramūnas Antanaitis, Dovilė Malašauskienė, Mindaugas Televičius, Vida Juozaitienė, Henrikas Žilinskas, Walter Baumgartner

**Affiliations:** 1 Large Animal Clinic, Veterinary Academy, Lithuanian University of Health Sciences, LT-47181 Kaunas, Lithuania; dovile.malasauskiene@lsmuni.lt (D.M.); mindaugas.televicius@lsmuni.lt (M.T.); henrikas.zilinskas@lsmuni.lt (H.Ž.); 2 Department of Animal Breeding, Veterinary Academy, Lithuanian University of Health Sciences, LT-47181 Kaunas, Lithuania; vida.juozaitiene@lsmuni.lt; 3 University Clinic for Ruminants, University of Veterinary Medicine, A-1210 Vienna, Austria; walter.baumgartner@vetmeduni.ac.at

**Keywords:** precision dairy farming, milk progesterone, production, reproduction, automatic milking system

## Abstract

**Simple Summary:**

According to the literature, the at-line progesterone monitoring system Herd Navigator^TM^ (Lattec I/S, Hillerød, Denmark) was used in combination with a DeLaval milking robot (DeLaval Inc., Tumba, Sweden). It works automatically and provides real-time physiological information about lactating dairy cows. For making farm-management decisions, it is not only a novel tool for scientific research, but also a mechanism for improving productivity, food safety, animal well-being, the environment, and the public perception of the dairy industry. It has been hypothesized that the progesterone concentration determined by the at-line milk analysis system and changes in its dynamics correlate with the parity, reproductive status, and milk yield of cows. The aim of the current study was to evaluate relative at-line milk progesterone (mP4) dynamic changes, according to the parity and status of reproduction, and to estimate the relationship with productivity in dairy cows. Frequent automated mP4 sampling can help identify characteristics of mP4 dynamic changes associated with successful pregnancies, pregnancy losses, and potential differences in mP4 dynamics among parity groups, which have not been studied previously.

**Abstract:**

The aim of the current instant study was to evaluate relative at-line milk progesterone dynamic changes according to parity and status of reproduction and to estimate the relationship with productivity in dairy cows by at-line milk analysis system Herd Navigator^TM^. According to the progesterone assay, experimental animals were divided into three periods: postpartum, after insemination, and pregnancy. In the first stage of the postpartum period, progesterone levels in milk were monitored every 5 days. This period of reproductive cycle recovery was followed for 30 days (days 0–29). The second stage of the postpartum period (30–65 days) lasted until cows were inseminated. In the period (0–45 days) after cow insemination, progesterone levels were distributed according to whether or not cows became pregnant. For milk progesterone detection, the fully automated real-time progesterone analyzer Herd Navigator^TM^ (Lattec I/S, Hillerød, Denmark) was used in combination with a DeLaval milking robot (DeLaval Inc., Tumba, Sweden). We found that an at-line progesterone concentration is related to different parities, reproductive statuses, and milk yield of cows: the 12.88% higher concentration of progesterone in milk was evaluated in primiparous cows. The average milk yield in non-pregnant primiparous cows was 4.64% higher, and in non-pregnant multiparous cows 6.87% higher than in pregnant cows. Pregnancy success in cows can be predicted 11–15 days after insemination, when a significant increase in progesterone is observed in the group of pregnant cows.

## 1. Introduction

Milk progesterone (mP4) is considered the gold standard for the evaluation of reproduction status for research purposes [1]. Data for mP4 have been widely used to characterize ovarian activity and pregnancy status and to evaluate associations between progesterone (P4) levels around the time of artificial insemination (AI) and pregnancy [2]. Studying actual progesterone dynamic changes should improve our knowledge of the effects of profile features and can indicate the chances of insemination success. However, current routine methods performed in the laboratory for progesterone quantification usually need complicated facilities and equipment. According to Bruinjé et al. [3], characterizing complete P4 profiles from early postpartum period until pregnancy establishment in lactating dairy cows through manual milk sampling is labor-intensive, and hence, rarely done. Consistently measuring progesterone concentrations also adds value to identifying pregnancy losses associated with luteal regression [4]. Therefore, these methods are expensive, and special skills and knowledge are needed to interpret the obtained data when they are not visually conclusive [4]. A fully automated at-line progesterone analyzer, Herd Navigator (Lattec I/S, Hillerød, Denmark), which can be combined with a DeLaval milking robot (DeLaval Inc., Tumba, Sweden), has recently become available in the commercial market. Although it requires a considerable investment at the installation stage, which would be costly for small farms, this tool is profitable for large farms compared to the frequent manual collection of progesterone information [5]. An at-line sampler inside the milking robot automatically takes a representative sample of several milliliters of milk from an individual cow during milking. The sample is then submitted to the Herd Navigator^TM^, which measures progesterone using a dry-stick technique based on an immunoassay [6]. Using the Herd Navigator^TM^ data, significant differences in mP4 dynamic changes among primiparous and multiparous cows and among cows with different AI outcomes are observed. Wider adoption of this precision technology would certainly improve our understanding of the factors affecting the reproductive physiology of the modern dairy cow, but it would also help enhance fertility in dairy herds [3]. The main features of the Herd Navigator^TM^ system for reproductive management include monitoring of resumption of postpartum luteal activity and subsequent luteal phases, declaring imminent estrus based on cessation of a luteal phase, and estimating early nonpregnancy or pregnancy status, all based on mP4 dynamic changes [3].

Using this precision technology can improve our understanding of the factors affecting the reproductive physiology of the modern dairy cow, and informed decision making can improve fertility in dairy cows. In addition to assisting with reproductive management decisions, the assessment of frequent mP4 data generated by Herd Navigator^TM^ gives a new opportunity to evaluate parameters of luteal activity [7,8], such as mP4 levels at specific time points, and their associations with fertility. Given the considerable variability in luteal phase length in the modern dairy cow [3], evaluating the characteristics of luteal activity through mP4 profiles in cows that conceived and maintained a pregnancy would enhance the understanding of dairy cow fertility [3]. An at-line progesterone monitoring system that works automatically and provides real-time physiological information about lactating dairy cows for making farm-management decisions is not only a novel tool for scientific research, but also improves productivity, food safety, animal well-being, the environment, and the public perception of the dairy industry [4]. The Herd Navigator^TM^ has resulted in a reasonably accurate system, but more work is needed to validate this system. Taking advantage of at-line progesterone monitoring will also encourage researchers to optimize the productive process in lactating dairy cows. Undoubtedly, disseminating its significance to the public and society will hasten the approval and adoption of this technology [5].

According to this, we hypothesized that mP4 concentration data generated through an automated Herd Navigator^TM^ system to determine changes in its dynamics correlate with the number of lactations, reproductive status, and milk yield of cows. This study aimed to provide information in progesterone dynamic changes determined by the at-line milk analysis system Herd Navigator^TM^, according to parity and status of reproduction and to estimate the relationship with productivity in dairy cows.

## 2. Materials and Methods

### 2.1. Location and Experimental Design

The study was carried out on a dairy farm in the eastern region of Europe in the northern part of Lithuania. Lithuanian Black and White dairy cows were selected. The study was performed on 624 dairy cows from a herd of 855 cows. The cows were kept in a loose housing system and fed a total mixed ration (TMR) throughout the year at the same time, balanced according to their physiological needs. Cows were fed a TMR consisting of 30% corn silage, 10% grass silage, 4% grass hay, 50% grain concentrate mash, and 6% of mineral mixture. Diets were formulated accordingly to meet or exceed the requirements of a 550 kg Holstein cow producing 35 kg/d. Composition of ration: dry matter (DM) (%) 48.8; neutral detergent fiber (% of DM) 28.2; acid detergent fiber (% of DM) 19.8; nonfiber carbohydrates (% of DM) 38.7; crude protein (% of DM) 15.8; neto energy for lactation (Mcal/kg) 1.6. Feeding took place every day at 06:00 and 18:00. The cows were milked two times per day through a parlor system at 05:00 and 17:00. The average weight of the cows was 550 kg +/−45 kg. Cows were housed in ventilated free-stall barns. The average energy corrected milk yield (4.2% fat, 3.5% protein) in 2019 was 9500 kg per cow and year. During the study, contact with the animals was minimal, and animal welfare issues were avoided.

### 2.2. Measurements

The Herd Navigator™ is programmed to collect milk samples automatically and analyze mP4 in individual cows through a dry-stick biosensor technology and enzyme immunoassay, based on a bio-model that establishes frequency and quantification of mP4 samples. The system modulated the frequency of assays at an average of six to seven progesterone analyses per cycle, according to the postpartum period and the stage of the estrous cycle. After this, the analyzer sent the data to a user interface. For the estrous cycling cattle, a heat alert was displayed as soon as the progesterone level dropped below 5 ng/mL. Together with the heat alert, the algorithm also displayed the percentage of success of prospective artificial insemination according to the span of the previous luteal phase and the dynamics of the decrease in progesterone values.

The mP4 concentration in milk samples started at first day postpartum and were taken, on average, every 5 d, until a pregnancy was declared. To reduce the random variation and differences in batches of sticks and reagents, raw (actual) mP4 concentrations were adjusted to smoothed values based on a standardized method to control for outliers expected in the serial sampling system, as described by Friggens and Chagunda [9].

### 2.3. Animals and Experimental Conditions

A total of 331 primiparous and 293 multiparous cows (2+ lactations) were selected.

According to cows’ reproductive status, the cows were classified as belonging to the nine groups listed in Table 1. During this data acquisition period, cows were subdivided into groups of target progesterone levels.

According to the progesterone assay, experimental animals were divided into three periods: postpartum, after insemination, and pregnancy. In the first stage of the postpartum period, progesterone levels in milk were monitored every 5 days. This period of reproductive cycle recovery was followed for 30 days (days 0–29). The second stage of the postpartum period (30–65 days) lasted until cows were inseminated. In the period (0–45 days) after cow insemination, progesterone levels were distributed according to whether or not cows became pregnant.

Additionally, pregnancies were examined on day 46 and confirmed 60 d after insemination with a digital diagnostic ultrasound scanner (Draminski iScan, Draminski S.A., Olsztyn, Poland) at a frequency of 7.5 MHz using a linear rectal transducer.

The study was carried out in compliance with EU legislation. All procedures complied with the criteria given by the Lithuanian animal welfare regulations (No. B1-866, 2012; No. XI-2271, 2012) and decree of the director of the State Food and Veterinary Service, the Republic of Lithuania No. B6-(1.9)-855, 2017.

### 2.4. Data Analysis and Statistics

The statistical analysis of data was performed using the SPSS 20.0 (SPSS Inc., Chicago, IL, USA) program package. Using descriptive statistics, normal distributions were assessed by Kolmogorov–Smirnov test. The results were expressed as the mean ± standard error of the mean (M ± S.E.M.). The Pearson’s correlation (*r*) was calculated to define the statistical relationship between milk progesterone, highest milk yield (HMY), and average milk yield (AMY). Differences in the mean values of normally distributed variables were analyzed using Student’s *t*-test (the independent samples *t*-test was used for evaluation of differences between primiparous and multiparous cows, and between cows that did become pregnant and those that did not). A probability of less than 0.05 was considered significant (*p* < 0.05).

A binary logistic regression technique was performed using pregnancy as the dependent variable (where 1 denotes pregnancy and 0 denotes its absence) to predict the relationship with AMY and milk progesterone levels in cows. AMY values between groups differed in a statistically significant way from 31 to 35 days after insemination, and progesterone values in milk from 11 to 15 days after insemination (Table 2. Predictors for logistic regression were considered class variables in the analyses. Values of milk progesterone, estimated 11–15 days after insemination, were divided into two groups: ≤14.73 ng/mL (class 0) and >14.73 ng/mL (class 1). Values of AMY, estimated 31–35 days after insemination, were divided into two groups: ≤19.04 kg (class 0) or >19.04 kg (class 1); milk progesterone values were also divided into two groups: ≤38.35 ng/mL (class 0) and >38.35 ng/mL (class 1). The classification threshold for these indicators was selected based on its arithmetic mean. Estimates and Wald 95% limits were used to calculate odds ratios (ORs) and 95% confidence intervals (CIs).

## 3. Results

### 3.1. Changes in the Concentration of Milk Progesterone According to Status of Reproduction in Dairy Cows

**Figure 1:**
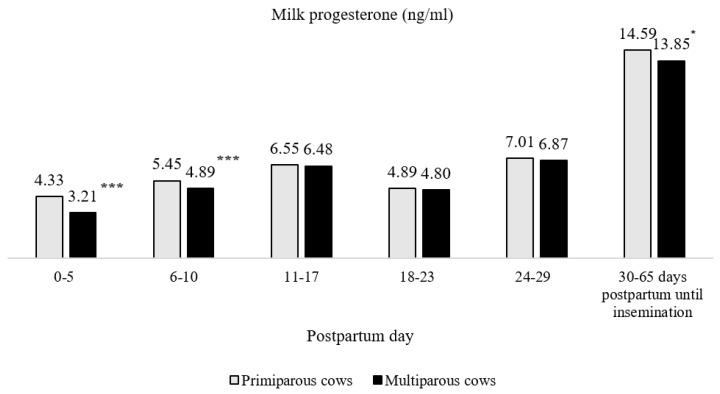
Changes in the concentration of milk progesterone during the postpartum period (* *p* < 0.05, *** *p* < 0.001—average values of progesterone in milk between primiparous and multiparous cows are statistically significantly different).

In the first five days of the postpartum period (Figure 1), the progesterone concentration in the cows’ milk ranged from 3.21 to 4.33 ng/mL in multiparous and primiparous cows, respectively. In the subsequent period (6–10 days), progesterone concentration increased to 4.89 and 5.45 ng/mL for multiparous and primiparous cows, respectively. In the third period (11–17 days postpartum), progesterone levels increased significantly to an average of 6.48 and 6.55 ng/mL in multiparous and primiparous cows, respectively.

We also determined that the highest progesterone concentration in primiparous cows ranged from 1.08% (11–17 days postpartum) to 34.89% (0–5 days postpartum; *p* < 0.001) higher than that in cows of the second and higher parity. From 30 to 65 days postpartum until insemination, the concentration of milk progesterone in primiparous cows was 5.35% higher (*p* < 0.05) than that in the second and higher-parity cows.

**Figure 2:**
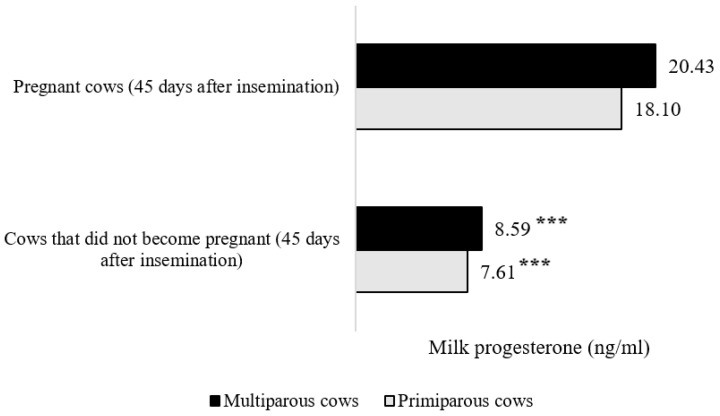
Changes in the level of milk progesterone (ng/mL) after insemination and during the pregnancy period (***—average values of progesterone in milk between did-become pregnant and did-not-become pregnant primiparous and multiparous cows with different superscripts differ significantly at *p* < 0.001).

The lowest progesterone concentrations in the milk of all cows were estimated during the first 5 postpartum days and between 18 and 23 days after calving. The increase of milk progesterone concentration was evaluated on days 24–29 after calving, and it was 1.34–1.35 times higher (*p* < 0.05) than the milk progesterone concentration on days 18–23 postpartum and 1.62–2.14 times (*p* < 0.001) higher than that on days 0–5 postpartum. In the 30–65 days after calving, the level of milk progesterone was 2.02–2.08 times higher than that on 24–29 days postpartum (*p* < 0.001). After insemination, the level of progesterone in milk increased by 10.77–22.54% compared with that in cows on days 30–65 after calving (*p* < 0.01). A higher (12.88%) concentration of progesterone in milk was evaluated in the primiparous cow group (*p* < 0.01) compared with that in cows of the second and higher parity group. In pregnant cows, milk progesterone within 0–45 days after insemination was 23.88% (in primiparous cows) and 32.54% (in multiparous cows) higher than that in non-pregnant cows (*p* < 0.001). These data are presented in Figure 2.

### 3.2. Changes in Milk Productivity of Cows According to Status of Reproduction

The AMY in primiparous cows increased 2.30–2.40 times from 0–5 days postpartum to 30–65 days, and HMY increased 2.07–2.08 times (*p* < 0.001). A slowdown in productivity growth was observed during two periods: 6–11 days postpartum and 18–23 days (Table 2).

**Figure 3:**
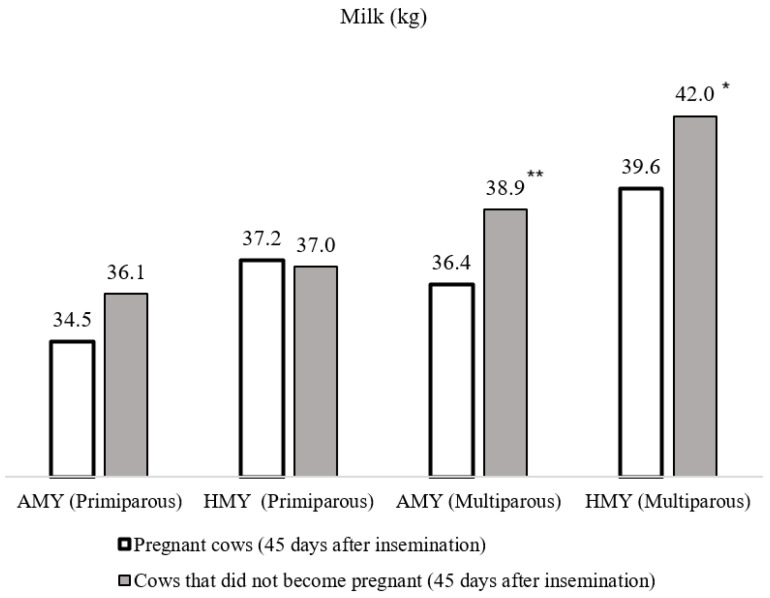
The milk yield (kg) of cows 0–45 days *p* < 0.05, ** *p* < 0.001 after insemination (*—average values of progesterone in milk between primiparous and multiparous cows are statistically significantly different).

Non-pregnant cows within 0–45 days after the insemination period were more productive (Figure 3). The AMY in non-pregnant primiparous cows (36.12 ± 1.72 kg) was 44.64% higher, and in non-pregnant multiparous cows (38.91 ± 1.69 kg), it was 6.87% higher (*p* < 0.01) than in pregnant cows. We estimated a significantly lower HMY (6.06%, *p* < 0.05) in pregnant multiparous cows, compared with cows that did not become pregnant (39.65 ± 0.62 kg).

### 3.3. Relationship of Progesterone in Milk with Productivity of Cows According to Status of Reproduction

Correlation coefficients between the progesterone and milk yield of cows are presented in Table 3. The data indicate that cows’ AMY and HMY were positively associated with milk progesterone concentration; a statistically significant relationship was found at 18–23 days postpartum (*r* = 0.324–0.346; *p* < 0.05). The significant coefficient of correlation was estimated between AMY and milk progesterone in pregnant cows 0–45 days after insemination (*r* = 0.251; *p* < 0.05).

Logistic regression analysis showed that at 31–35 days after insemination, cows with higher progesterone levels in their milk had a statistically significant higher probability of pregnancy success (OR = 8.53; 95% CI = 1.616–45.061; *p* = 0.022) compared with cows with lower progesterone levels in their milk. Milk yields in cows did not have a statistically significant effect in predicting the likelihood of their pregnancy.

**Figure 4:**
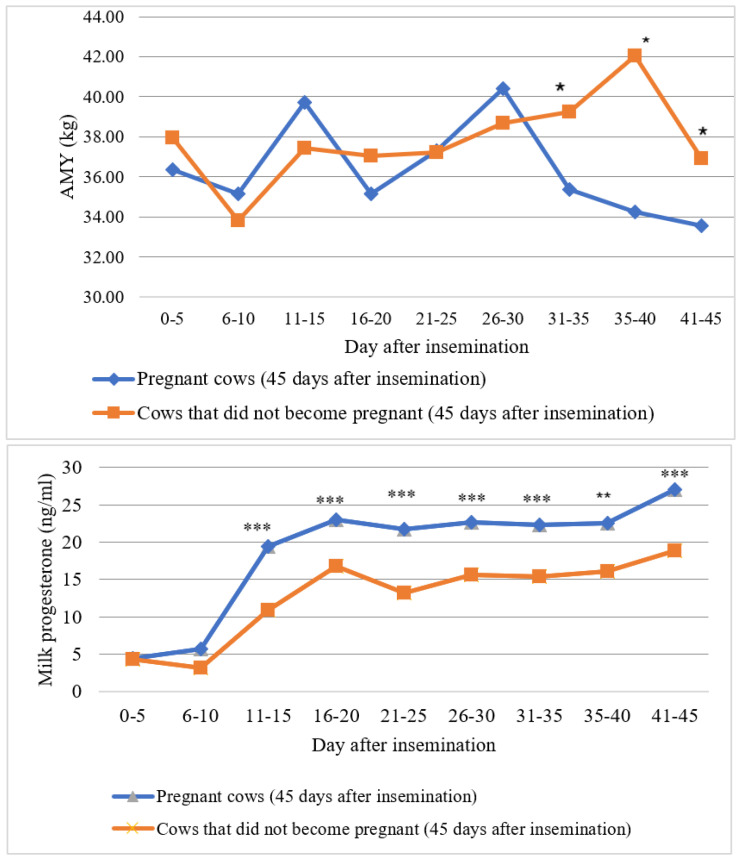
Changes in milk yield and progesterone in cows that became pregnant and did not become pregnant after insemination (* *p* < 0.05, ** *p* < 0.01, *** *p* < 0.001—average values between did-become-pregnant and did-not-become-pregnant cows are statistically significantly different).

The study showed that, depending on the concentration of progesterone in milk, pregnancy success in cows can be predicted 11–15 days after insemination, when a significant increase in progesterone is observed in the group of pregnant cows (OR = 7.43; 95% CI = 1.778–31.040; *p* = 0.006) compared with non-pregnant cows (Figure 4).

## 4. Discussion

This study investigated at-line progesterone dynamic changes according to milk yield, lactation number, and status of reproduction in dairy cows. The results show and confirm that progesterone concentration within pregnant cattle was higher than that in other investigated cattle groups, which coincides with the results reported by Hommeida et al. [7]. Larson et al. [8] noted that progesterone concentrations 5–10 days after insemination were higher in pregnant cattle. In addition to being more sensitive to metabolic changes (e.g., negative energy balance) in the early postpartum period than multiparous cows, primiparous cows are more likely to develop uterine diseases, which are known factors affecting resumption of postpartum cyclicity [9]. We found that, 31–35 days after insemination, cows with higher progesterone levels in their milk had a statistically significant higher probability of pregnancy success. According to Berger et al. [10], the luteal area and progesterone concentration were greater in pregnant cattle compared with those in open cattle. The overall mean milk progesterone concentration in pregnant cattle did not significantly differ from that in non-pregnant animals, but there was a strong quadratic relationship between both low and high milk progesterone concentrations associated with reduced conception rates. The pregnant animals did not experience an increase in the pulsatile release of PGF2α, and the CL was maintained for a further 200 days of pregnancy [11], and for this failure, the mechanism was the reason of early embryonic loss [12]. Herzog et al. [13] reported that embryonic loss did not have any influence on the plasma concentration progesterone or the luteal area, which was similar between pregnant cattle and cattle with apparent embryonic loss. According to Gomez-Seco et al. [14], in the pregnant group, the general correlation (from day 0 to 23) was lower than that in non-pregnant cattle, although, during the developing phase, this value was similar in both groups. Lactating dairy cows have subnormal progesterone concentrations, as a result of a higher liver blood flow, which increases the rate of progesterone catabolism [15]. This subnormal progesterone concentration allows for an increased frequency of LH secretion impairing follicular and oocyte competence, and embryonic survival [16]. According to the results of the current study in the period 6–10 days, progesterone concentration increased to 4.89 and 5.45 ng/mL in multiparous and primiparous cows, respectively. The faster rise in mP4 from in the first days after calving was associated with improved pregnancy maintenance. In addition, higher mP4 after d 4 relative to AI was associated with greater trophoblast length at later stages, greater uterine concentrations of interferon-tau, and reduced pregnancy losses [14]. Our results suggest that improved conception is associated with high mP4 beyond day 10, regardless of whether the pregnancy is sustained or lost.

The results of our study show that 12.88% higher concentration of progesterone in milk was evaluated in primiparous cows. The results of our study show that multiparous cattle had a lower progesterone concentration, which coincides with the findings of Pineyrua et al. [17], who found that the mean concentrations of postpartum mP4 were different between multiparous and primiparous cattle. Pineyrua et al. [17] and Caixeta et al. [18] reported limited data to support our hypothesis that metabolic and hormonal changes during the first 4 weeks of lactation possess negative carryover effects on the reproductive performance of Holstein and Jersey dairy cattle later in the lactation period. The higher mP4 levels in primiparous cows than in multiparous cows were expected, as a higher milk yield in multiparous cows would be associated with greater dry matter intake and, consequently, accelerated metabolic clearance of mP4, decreasing its peripheral concentrations. Multiparous cows reportedly required greater doses of exogenous PGF2α than primiparous cows to achieve a similar luteolysis rate, suggesting potential differences in corpus luteum (CL) responses to PGF2α (greater occurrence of incomplete CL regression in multiparous than in primiparous cows) [18]. Primiparous cows had different mP4 dynamic changes (greater levels and more rapid increase early post-AI) than multiparous cows [3]. Recent reports in a research herd indicate that differences in ovarian function exist between primiparous and multiparous cows [15]. Frequent automated mP4 sampling can help identify characteristics of mP4 dynamic changes associated with successful pregnancies, pregnancy losses, and potential differences in mP4 dynamics among parity groups, which have not been studied previously.

We found that non-pregnant cows were more productive compared to pregnant cows. According to Penasa et al. [19], pregnancy was shown to exert a significant effect on milk yield. Therefore, some researchers have proposed an antagonistic relationship between milk yield and fertility [20,21]. Many studies have addressed this controversial topic with inconsistent approaches and results. Some found negative associations [22], whereas others reported a positive relationship [23]. We found that milk yield was positively associated with milk progesterone concentration at 18–23 days postpartum.

## 5. Conclusions

According to our study results, we can suggest that an at-line progesterone concentration determined by the milk analysis system, Herd Navigator^TM^, and changes in its dynamics correlate with different parities, reproductive statuses, and the milk yield of cows: a higher concentration of progesterone in milk was evaluated in primiparous cows, and non-pregnant cows were found to be more productive.

Pregnant cows 31–35 days and 11–15 days after insemination have higher milk progesterone levels (23.88% (in multiparous cows) and 32.54% (in primiparous cows) than that in non-pregnant cows), which positively associated with a successful pregnancy. Pregnancy success in cows can be predicted 11–15 days after insemination, when a significant increase in progesterone is observed in the group of pregnant cows.

## Figures and Tables

**Table 1:** Creation of experimental groups.

Group Number/Days Postpartum	Number of Cows	
	Multiparous	Primiparous
(1) 0–5	20	18
(2) 6–10	17	19
(3) 11–17	19	22
(4) 18–23	18	21
(5) 24–29	20	23
(6) 30–65 days postpartum until insemination	47	53
(7) 0–45 days after insemination	30	37
(8) Cows that did not become pregnant (45 days after insemination)	44	52
(9) Pregnant cows (45 days after insemination)	78	86

**Table 2:** The milk yield of cows in the postpartum period until insemination. AMY—average milk yield; HMY—highest milk yield. *—average values of AMY or HMY between primiparous and multiparous cows are statistically significantly different at *p* < 0.05.

Postpartum Day	AMY		HMY	
	Primiparous Cows	Multiparous Cows	Primiparous Cows	Multiparous Cows
0–5	14.6 ± 0.91	15.0 ± 0.87	17.9 ± 0.99	18.4 ± 1.03
6–10	15.4 ± 0.62	16.9 ± 0.68 *	19.8 ± 0.98	19.9 ± 1.09
11–17	22.4 ± 1.28	22.8 ± 1.11	28.5 ± 1.38	32.1 ± 1.62 *
18–23	23.5 ± 1.19	25.1 ± 1.14 *	27.8 ± 0.93	33.0 ± 1.45 *
24–29	31.6 ± 1.92	31.8 ± 1.90	34.2 ± 2.00	37.3 ± 1.92 *
30–65 days postpartum until insemination	35.1 ± 1.84	34.5 ± 1.98	37.2 ± 1.86	38.1 ± 1.94

**Table 3:** Correlation of progesterone in milk with milk yield of cows (* *p* < 0.05—Pearson’s correlation is statistically significant).

Period/Status of Reproduction			Coefficients of Correlation	
			AMY	HMY
**Postpartum Period**	0–30 days postpartum	0–5 days	0.142	0.072
		6–10 days	0.162	0.130
		11–17 days	0.261	0.207
		18–23 days	0.346 *	0.324 *
		24–29 days	0.212	0.275
	30–65 days postpartum until insemination		0.092	0.048
**Period after Insemination**	0–45 days after insemination	Became pregnant	0.251 *	0.104
		Did not become pregnant	0.082	0.066
